# Impact of oliceridine versus sufentanil on postoperative nausea and vomiting in patients undergoing thyroid surgery: a prospective, double-blind, randomized controlled trial

**DOI:** 10.1080/07853890.2026.2690695

**Published:** 2026-06-24

**Authors:** Feng Liu, Wang Shen, Dan Su, Zhaoming Guan, Yi Zhang, Siyu Li, Qisong Yuan, Yupeng Zhao, Jixiong Sun, Hongwei Fang, Longqiu Yang

**Affiliations:** ^a^ Department of Anesthesiology, Shanghai East Hospital, School of Medicine, Tongji University, Shanghai, China; ^b^ Department of Pain Medicine, Renji Hospital, School of Medicine, Shanghai Jiao Tong University, Shanghai, China

**Keywords:** Oliceridine, sufentanil, postoperative nausea and vomiting, thyroid surgery, opioid-related adverse events

## Abstract

**Purpose:**

Postoperative nausea and vomiting (PONV) is a common complication following thyroid surgery, often exacerbated by opioid use. Oliceridine, a novel G protein-biased μ-opioid receptor agonist, may reduce opioid-related adverse events. This study aimed to compare the impact of oliceridine versus sufentanil on the incidence and severity of PONV in patients undergoing thyroid surgery.

**Patients and methods:**

In this prospective, double-blind, randomised controlled trial conducted between May 2025 and February 2026, 232 patients scheduled for thyroid surgery were randomly assigned to receive either oliceridine or sufentanil for intraoperative analgesia. The primary outcome was the incidence of PONV during the first 48 h postoperatively. Secondary outcomes included PONV severity, need for rescue anti-emetics, postoperative pain scores, recovery quality, and other adverse events.

**Results:**

The incidence of PONV within 48 h postoperatively was significantly lower in the oliceridine group [13/107 (12%)] compared with the sufentanil group [31/110 (28%)] (OR = 0.35, 95% CI: 0.17–0.72, *p* = 0.006). Postoperative pain scores, rescue analgesia requirements, and Quality of Recovery-15 scores were comparable between the two groups (*p* > 0.05). Besides, exploratory unadjusted analyses revealed fewer rescue anti-emetics: O group 8/107 (8%) *vs* S group 25/110 (23%) (OR = 0.27, 95% CI: 0.12–0.64, *p* = 0.002); and less abdominal distension: O group 4/107 (4%) *vs* S group 19/110 (17%) (OR = 0.19, 95% CI: 0.06–0.57, *p* = 0.001).

**Conclusion:**

For young ASA I–II patients undergoing thyroid surgery, oliceridine yields adequate postoperative analgesia and lower PONV rates versus sufentanil. Additional trials involving high-intensity surgical procedures are needed to confirm consistent equivalence.

## 1. Introduction

Postoperative nausea and vomiting (PONV) is a frequent and clinically significant complication following thyroid surgery, with incidence rates ranging from 20% to 60% [1,2]. Key predictors, as per the Apfel scoring system, include female gender, non-smoking status, history of PONV or motion sickness, and postoperative opioid use [3]. PONV impairs early recovery, diminishes patient satisfaction, and can lead to serious sequelae like dehydration, electrolyte imbalances, and increased wound stress, making its effective prevention a priority [4,5].

Postoperative pain management often requires opioids, which are themselves a key risk factor for PONV, creating a therapeutic challenge [6]. This underscores the need for analgesics that provide effective pain relief while minimising adverse effects. Oliceridine is a novel G protein-biased μ-opioid receptor agonist. Its mechanism involves selective activation of G protein pathways responsible for analgesia, while downregulating β-arrestin recruitment [7]. The β-arrestin pathway is strongly associated with opioid-induced side effects [8]. Compared to conventional opioids like morphine, oliceridine induces substantially less β-arrestin signalling. This profile suggests it may deliver potent analgesia with potentially improved opioid-induced side effects, specifically a reduced propensity for inducing PONV.

Despite this promising rationale, clinical evidence on oliceridine’s effect on PONV in specific surgical contexts remains limited. Patients undergoing thyroid surgery represent a population at inherently high risk. Currently, no study has specifically investigated oliceridine’s efficacy in modulating PONV outcomes in this vulnerable group.

Therefore, this study aims to compare the impact of oliceridine versus sufentanil, a commonly used potent opioid, on the incidence and severity of PONV in patients undergoing thyroid surgery. We hypothesised that oliceridine would reduce the incidence of PONV compared with sufentanil in patients undergoing thyroid surgery.

## 2. Materials and methods

### 2.1. Ethics and patients

This prospective, double-blind, randomised controlled trial was carried out in a Chinese tertiary hospital from May 2025 to February 2026. It received ethical approval from the Shanghai East Hospital Medical Ethics Committee (approval number: 2025YS-086) and was registered with the Chinese Clinical Trial Registry (ChiCTR2500101375). All procedures were performed in line with the Declaration of Helsinki. Every participant gave written informed consent before being enrolled. The study also followed the Consolidated Standards of Reporting Trials (CONSORT) reporting guidelines.

The inclusion criteria are as follows: (1) aged 18–75 years, (2) body mass index (BMI) 18–35 kg/m^2^, (3) diagnosed with thyroid nodules or tumours by a senior surgeon and scheduled for surgical removal, (4) the American Society of Anaesthesiologists (ASA) Physical Status I-II. The exclusion criteria are as follows: (1) dyspnoea or tracheal compression, (2) obstructive sleep apnoea syndrome, (3) history of neurological diseases such as stroke or epilepsy, (4) poor-controlled hypertension, diabetes, cardiac diseases, hepatic or renal impairment and malignant tumour, (5) chronic opioid use or suffering from painful diseases, (6) allergy to the drugs used in this study, (7) psychiatric disorders that prevent cooperation with the study, (8) pregnant or breastfeeding, (9) participation in other clinical trials.

### 2.2. Randomisation and blinding

Randomisation was carried out using a sequence created by R software (version 4.2.1; http://www.R-project.org), with a 1:1 assignment to either the oliceridine group (Group O) or the sufentanil group (Group S). To minimise allocation bias, an independent nurse who was not otherwise involved in the study generated and kept the allocation sequence. Concealment was ensured by sequentially numbered, sealed, opaque envelopes that were prepared in advance. These envelopes were stored securely and could not be accessed by any personnel involved in recruitment, data collection, or clinical care until the moment of allocation. In a medical emergency requiring immediate knowledge of the intervention, the attending physician was allowed to open the envelope to break the blind.

For double-blinding, once the patient arrived in the operating room, an anaesthesia nurse (not part of the study team) opened the envelope to learn the group assignment. A senior anaesthesiologist, who was unaware of the group allocation, wrote a medication order that included both oliceridine and sufentanil. Based on this order and the actual group assignment, the anaesthesia nurse prepared the medication—containing either oliceridine or sufentanil together with the usual routine drugs—so that the senior anaesthesiologist never knew which specific drug was given. After surgery, two independent anaesthesiologists, both blinded to group assignment, jointly assessed all study endpoints. Throughout the trial, blinding was maintained for patients, surgeons, intraoperative anaesthesia providers (who managed anaesthesia but did not prepare drugs), postoperative caregivers, and the statistician who performed the final analysis.

### 2.3. General anesthesia procedure

All patients followed standard preoperative fasting and abstention from fluids. Upon entering the operating room, oxygen was administered *via* nasal cannula at a flow rate of 2 L/min. Patients received standard monitoring. Ten minutes prior to anaesthesia induction, dexamethasone 5 mg and ondansetron 4 mg were administered intravenously for prophylaxis against PONV. For anaesthesia induction, patients in Group S received sufentanil (Yichang Renfu Pharmaceutical Co., Ltd., Yichang, Hubei, China) at 0.3 μg/kg, while those in Group O received oliceridine (Jiangsu Enhua Pharmaceutical Co., Ltd., Xuzhou, Jiangsu, China) at 0.06 mg/kg. This was followed sequentially by etomidate at 0.2 mg/kg and rocuronium at 0.6 mg/kg. Subsequently, mask-assisted ventilation was initiated, and upon meeting the criteria for tracheal intubation, mechanical ventilation was established *via* an endotracheal tube. Ventilation parameters were adjusted to a tidal volume of 6–8 mL/kg, respiratory rate of 12–16 breaths/min, inspiratory-to-expiratory ratio of 1:2, and FiO_2_ of 60%, with the goal of maintaining P_ET_CO_2_ between 35 and 40 mmHg.

Anaesthesia was maintained using a combined intravenous and inhalation technique. Propofol was continuously infused at 4–6 mg·kg^−1^·h^−1^, supplemented by intermittent inhalation of 0.6%–1.5% sevoflurane. For intraoperative analgesia, patients in group S received an additional dose of sufentanil 0.15 μg/kg every 15 min, while group O received an additional dose of oliceridine 0.03 mg/kg every 15 min. Heart rate and blood pressure were maintained within 20% of baseline values by adjusting the propofol infusion rate or administering vasoactive agents such as ephedrine or phenylephrine as needed. Intermittent doses of rocuronium were given to maintain muscle relaxation.

At the conclusion of surgery, all patients received sugammadex for reversal of neuromuscular blockade. Once patients met the criteria for extubation, the tracheal tube was removed, and they were transferred to the post-anaesthesia care unit (PACU) for continued observation.

### 2.4. Postoperative management

Upon transfer to the ward, all patients were prescribed intravenous flurbiprofen axetil (50 mg per dose, once daily). Rescue analgesia with oxycodone hydrochloride tablet 10 mg (not to exceed three times per day) was given to treat postoperative pain with a score ≥4 on an 11-point numerical rating scale (NRS, 0 = no pain and 10 = the most severe pain). For rescue anti-emetics, intravenous ondansetron 4 mg was administered if required.

### 2.5. Outcome measurement

The primary outcome was the incidence of PONV—including nausea, retching, and vomiting—during the patient’s stay in the PACU, as well as within 0–24 h and 24–48 h postoperatively. Nausea referred to a subjective sensation of having the desire to vomit, while retching was characterised by involuntary gastric movements without the expulsion of stomach contents. Vomiting was defined as the forceful oral expulsion of gastric contents.

Secondary outcomes are as follow: (1) severity of PONV, which was categorised as mild (not hindering routine activities such as personal hygiene, dressing, or walking), moderate (occasionally interfering with daily functioning), or severe (severely limiting the ability to perform daily activities or involving three or more episodes of vomiting) [9], (2) need for rescue anti-emetics after surgery, (3) severity of postoperative pain, which was assessed with NRS [10], (4) need for rescue analgesia after surgery, (5) intraoperative data, which included surgical type, pathology, surgical time, opioids consumption and morphine equivalent dose (Sufentanil 1 μg equals 1 mg morphine equivalent approximately [11,12]; oliceridine 0.2 mg equals 1 mg morphine equivalent approximately [13–15], estimated infusion volume, estimated blood loss, incidence of hemodynamic events requiring intervention (hypertension, hypotension, tachycardia and bradycardia), extubation time (the time from the end of surgery to extubation), and time in PACU, (6) other postoperative adverse events, including haemorrhage or haematoma, nerve injury, abdominal distension, respiratory depression, dizziness or headache, nightmare, and drowsiness, (7) the Quality of Recovery-15 (QoR-15) score at 1 week and 2 weeks postoperatively [16].

### 2.6. Statistical analysis

Sample size was calculated using PASS 13.0 (NCSS, Kaysville, UT, USA) based on the incidence of PONV. In our preliminary study, the 48 h PONV incidence was 35% in Group S (*n* = 20) and 17% in Group O (*n* = 23). To detect this difference with α = 0.05 and β = 0.2, we needed 93 patients per group. Accounting for a possible 15% dropout rate, we targeted at least 107 patients per group.

We performed per-protocol analyses including all patients who were allocated with primary outcome data available. Best-worst case sensitivity analysis was conducted to assess the influence of missing primary data. The normal distribution of the variables was examined using the Kolmogorov-Smirnov test. Continuous data were presented as mean ± standard deviation and compared using the unpaired, 2-tailed *t* test if distributed normally. Categorical variables were reported as number (%) and compared using *χ^2^* or Fisher exact test, as appropriate. The interventional effect of the two groups was analysed further using odds ratio (OR) or mean difference with 95% confidence intervals (CI). Subgroup analysis were performed to assess the consistency of the treatment effect on the primary outcome. Interaction between treatment and subgroup variables was tested by including the interaction term in the model (post hoc exploratory with no multiplicity adjustment). Two-sided *p* < 0.05 was considered to be statistically significant. We completed statistical analyses based on SPSS 26.0 (SPSS, Chicago, IL, USA).

## 3. Results

### 3.1. Demographic and baseline characteristics

**Figure 1.**
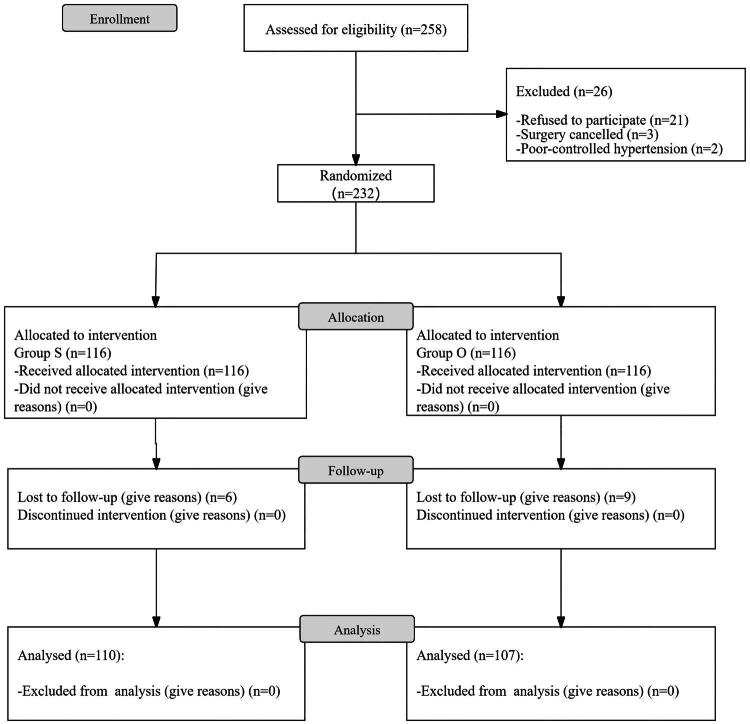
Study flow diagram. Six patients in Group S and 9 patients in Group O were lost to follow-up due to unsuccessful post-discharge contact. S, sufentanil; O, oliceridine.

A total of two hundred and fifty-eight patients from a tertiary hospital in China were enrolled in this study (Figure 1). Twenty-one patients refused to participate in the research. The surgeries for three patients were canceled. Two patients had poorly controlled hypertension. Eventually, two hundred and thirty-two patients were divided into two groups: Group S (*n* = 116) and Group O (*n* = 116). All randomised patients strictly received the allocated administration as assigned, with no protocol deviations. At follow-up, the primary outcome measure was not obtained for 6 patients in Group S and 9 patients in Group O, all of whom could not be contacted after hospital discharge; these patients were not included in the final analysis. The demographic data in terms of age, gender, height, weight, body mass index (BMI), ASA classification, occupation status, smoking status, history of PONV or motion sickness, total Apfel score, comorbidities, and preoperative QoR-15 score were comparable in both groups (*p* > 0.05) (Table 1).

**Table 1.** Demographic and baseline characteristics.

	Group O (*n* = 107)	Group S (*n* = 110)	Mean difference or odds ratio (95% CI)	*P* value
Age (y)	42.6 ± 14.8	41.8 ± 13.1	0.85 (−2.89 to 4.59)	0.653
Gender (male/female)	21/86	27/83	0.75 (0.39–1.43)	0.456
Height (m)	1.61 ± 0.07	1.62 ± 0.07	−0.01 (−0.03 to 0.01)	0.570
Weight (kg)	62.1 ± 8.5	60.7 ± 7.6	1.49 (−0.67 to 3.65)	0.176
BMI (kg/m^2^)	23.9 ± 3.5	23.3 ± 3.6	0.66 (−0.28 to 1.62)	0.168
ASA classification (I/II)	78/29	85/25	0.79 (0.43–1.46)	0.548
Retiree	24 (22%)	22 (20%)	1.16 (0.60–2.23)	0.775
Current smoker	10 (9%)	12 (11%)	0.84 (0.35–2.05)	0.887
History of PONV or motion sickness	25 (23%)	29 (26%)	0.85 (0.46–1.58)	0.720
Total Apfel score^a^	1.94 ± 0.71	1.91 ± 0.64	0.03 (−0.15 to 0.22)	0.705
Comorbidities				
Hypertension	16 (15%)	18 (16%)	0.90 (0.43–1.88)	0.925
Diabetes	8 (7%)	7 (6%)	1.19 (0.41–3.45)	0.950
Cardiac diseases	3 (3%)	2 (2%)	1.56 (0.18–13.70)	0.680
QoR-15 score	130.7 ± 7.0	129.7 ± 7.7	0.99 (−0.98 to 2.96)	0.323

Data presented as mean ± standard deviation were compared using the unpaired, 2-tailed *t* test. Data presented as the number of patients (%) were compared using the Pearson *χ^2^* test or Fisher exact test. ^a^The total Apfel score is calculated based on gender, smoking status, and history of postoperative nausea and vomiting or motion sickness, without considering postoperative opioid analgesics temporarily. O, oliceridine; S, sufentanil; CI, confidence intervals; BMI, body mass index; ASA, American Society of Anaesthesiologists; PONV, postoperative nausea and vomiting; QoR-15, Quality of Recovery-15.

### 3.2. Intraoperative outcomes

Surgery type, pathology, surgical time, opioids consumption, estimated infusion volume, estimated blood loss, incidence of hemodynamic events requiring intervention, extubation time, and time in PACU were similar between the two groups (*p* > 0.05) (Table 2).

**Table 2.** Intraoperative data.

	Group O (*n* = 107)	Group S (*n* = 110)	Mean difference or odds ratio (95% CI)	*P* value
Surgical type				
Thyroid lobectomy	71 (66%)	78 (71%)	0.81 (0.46–1.43)	0.560
Total thyroidectomy^a^	36 (34%)	32 (29%)		
Pathology				
Benign	69 (64%)	73 (66%)	0.92 (0.53–1.59)	0.880
Malignant	38 (36%)	37 (34%)		
Surgical time (min)	82.1 ± 16.5	79.0 ± 14.6	3.08 (−1.08 to 7.25)	0.146
Opioids consumption				
Sufentanil (μg)	−	60.8 ± 8.0	−	−
Oliceridine (mg)	11.9 ± 1.5	−	−	−
Morphine equivalent dose^b^ (mg)	59.4 ± 7.3	60.8 ± 8.0	1.37 (−0.68 to 3.41)	0.188
Estimated infusion volume (mL)	791 ± 101	805 ± 95	−15 (−41 to 11)	0.268
Estimated blood loss (mL)	34.8 ± 10.3	35.5 ± 11.1	−0.69 (−3.56 to 2.19)	0.637
Incidence of hypertension	8 (7%)	11 (10%)	0.73 (0.28–1.90)	0.696
Incidence of hypotension	10 (9%)	12 (11%)	0.84 (0.35–2.05)	0.887
Incidence of tachycardia	13 (12%)	16 (15%)	0.81 (0.37–1.78)	0.776
Incidence of bradycardia	5 (5%)	7 (6%)	0.72 (0.22–2.40)	0.833
Extubation time (min)	9.9 ± 2.0	10.1 ± 2.1	−0.21 (−0.76 to 0.33)	0.442
Time in PACU (min)	35.4 ± 3.5	35.0 ± 2.9	0.44 (−0.42 to 1.30)	0.314

Data presented as mean ± standard deviation were compared using the unpaired, 2-tailed *t* test. Data presented as the number of patients (%) were compared using the Pearson *χ^2^* test. ^a^Regardless of whether a neck lymph node dissection was performed. ^b^Sufentanil 1 μg equals 1 mg morphine equivalent approximately; oliceridine 0.2 mg equals 1 mg morphine equivalent approximately. O, oliceridine; S, sufentanil; CI, confidence intervals; PACU, post-anaesthesia care unit.

### 3.3. Postoperative nausea and vomiting assessment

Primary outcome data were available for 107 patients in Group O and 110 in Group S. Postoperative nausea and vomiting occurred in 13 of 107 (12%) patients allocated to Group O, compared with 31 of 110 (28%) patients allocated to Group S (risk difference = −16%, OR = 0.35, 95% CI: 0.17–0.72, *p* = 0.006). Besides, best-worst case sensitivity analysis was conducted to assess the influence of missing primary data, as PONV information was not available for patients lost to follow-up. PONV rates remained consistently lower in the oliceridine group across both extreme assumptions.

**Figure 2.**
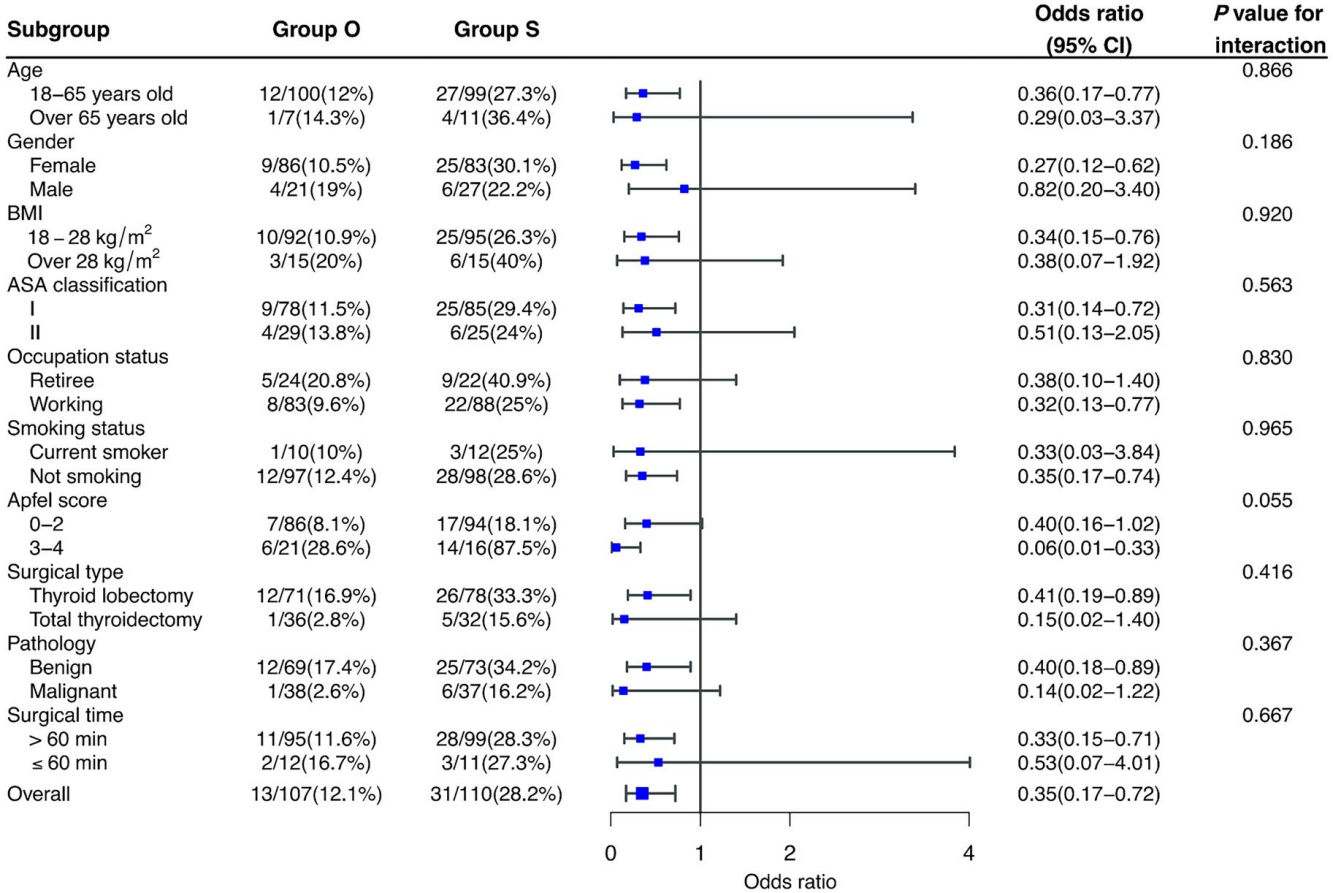
Subgroup analysis of postoperative nausea and vomiting (PONV). All subgroup analyses and interaction tests were post hoc and exploratory. The presented *P* values are nominal without adjustment for multiple comparisons. O, oliceridine; S, sufentanil; CI, confidence intervals; BMI, body mass index; ASA, American Society of Anaesthesiologists.

Most PONV episodes occurred within 24 h postoperatively (Table 3). In exploratory analyses, the difference between Group O and Group S remained consistent across the various subgroups (age, gender, BMI, ASA classification, occupation status, smoking status, Apfel score, surgical type, pathology and surgical time) (Figure 2).

**Table 3.** Assessment of postoperative nausea and vomiting.

	Group O (*n* = 107)	Group S (*n* = 110)	Odds ratio (95% CI)	*P* value
PONV during 0–48 h	13 (12%)	31 (28%)	0.35 (0.17–0.72)	0.006
PONV within different periods				
Within PACU	0	1 (1%)	−	−
0–24 h	13 (12%)	30 (27%)	0.37 (0.18–0.75)	0.009
24–48 h	1 (1%)	8 (7%)	0.12 (0–0.92)	0.036
PONV severity in the PACU				
Moderate	0	1 (1%)	−	−
PONV severity during 0–24 h				
Mild	9 (8%)	15 (14%)	0.58 (0.24–1.39)	0.220
Moderate	3 (3%)	8 (7%)	0.37 (0.10–1.43)	0.134
Severe	1 (1%)	7 (6%)	0.14 (0.01–1.00)	0.063
PONV severity during 24–48 h				
Mild	1 (1%)	7 (6%)	0.14 (0.01–1.00)	0.063
Moderate	0	1 (1%)	−	−
Need for rescue anti-emetics	8 (8%)	25 (23%)	0.27 (0.12–0.64)	0.002

Data were compared using the Pearson *χ^2^* test or Fisher exact test. O, oliceridine; S, sufentanil; CI, confidence intervals; PONV, postoperative nausea and vomiting; PACU, post-anaesthesia care unit.

As for the severity of PONV, one patient in Group S experienced moderate PONV in the PACU, which resolved after receiving ondansetron. During 0–24 h postoperatively, the majority of patients with PONV had mild symptoms, but one patient in Group O and seven patients in Group S experienced severe symptoms (all received ondansetron). During 24–48 h postoperatively, only one patient in Group O had mild PONV, while in Group S, seven had mild PONV and one had moderate PONV. Totally, the number of patients requiring rescue anti-emetics was lower in Group O [8/107 (8%)] than in Group S [25/110 (23%)] (OR = 0.27, 95% CI: 0.12–0.64, *p* = 0.002) (Table 3).

### 3.4. Other postoperative outcomes

There were no differences between the two groups in postoperative pain NRS scores (at PACU discharge, 24 h, and 48 h), the need for rescue analgesics, or QoR-15 scores (*p* > 0.05) (Table 4).

**Table 4.** Comparison of other secondary outcomes.

	Group O (*n* = 107)	Group S (*n* = 110)	Mean difference or odds ratio (95% CI)	*P* value
Pain NRS scores				
PACU discharge	1.42 ± 0.61	1.36 ± 0.74	0.06 (−0.13 to 0.24)	0.538
24 h	1.58 ± 0.73	1.52 ± 0.83	0.06 (−0.15 to 0.27)	0.583
48 h	1.03 ± 0.16	1.01 ± 0.17	0.02 (−0.03 to 0.06)	0.427
Need for rescue analgesics	3 (3%)	3 (3%)	1.03 (0.20–5.21)	1.000
Postoperative adverse events				
Abdominal distension	4 (4%)	19 (17%)	0.19 (0.06–0.57)	0.001
Respiratory depression	0	2 (2%)	−	−
Dizziness or headache	27 (25%)	33 (30%)	0.79 (0.43–1.43)	0.432
Nightmare	2 (2%)	1 (1%)	2.08 (0.18–48.36)	0.621
Drowsiness	13 (12%)	17 (15%)	0.76 (0.35–1.65)	0.481
QoR-15 scores				
1 week	133.3 ± 7.4	133.0 ± 8.1	0.25 (−1.82 to 2.33)	0.810
2 weeks	133.0 ± 8.6	133.3 ± 8.9	−0.33 (−2.67 to 2.01)	0.783

Data presented as mean ± standard deviation were compared using the unpaired, 2-tailed *t* test. Data presented as the number of patients (%) were compared using the Pearson *χ^2^* test or Fisher exact test. O, oliceridine; S, sufentanil; CI, confidence intervals; NRS, numerical rating scale; PACU, post-anaesthesia care unit; QoR-15, Quality of Recovery-15.

Group O [4/107 (4%)] had less postoperative abdominal distension than Group S [19/110 (17%)], (OR = 0.19, 95% CI: 0.06–0.57, *p* = 0.001), while there were no significant differences in other adverse events (*p* > 0.05) (Table 4). All adverse events were managed with symptomatic treatment. None of the patients in either group experienced surgical site haemorrhage or haematoma, nerve injury, or other surgery-related complications.

## 4. Discussion

In this prospective, double-blind, randomised controlled trial, we compared the effects of oliceridine with those of sufentanil on PONV outcomes in patients undergoing thyroid surgery. Our findings demonstrate that oliceridine significantly reduced the incidence of PONV during the first 48 h postoperatively, as well as the need for rescue anti-emetics. Importantly, the two groups showed comparable postoperative pain scores and rescue analgesia requirements, indicating that oliceridine provided equivalent analgesia to sufentanil.

In thyroid surgery, the incidence of postoperative nausea and vomiting is relatively high due to factors such as the majority of patients being young women, the necessity of intraoperative neck hyperextension, and the surgical stimulation of peripheral nerves in the neck [17]. Severe vomiting may lead to dehiscence of the neck incision, bleeding, or even compression of the airway [18]. Meanwhile, sufentanil is commonly used as a potent analgesic in thyroid surgery, with its analgesic efficacy being approximately 1000 times that of morphine [11,12]. However, sufentanil is prone to causing various opioid-related adverse reactions, necessitating cautious clinical use [19]. Oliceridine, a novel G protein-biased μ-opioid receptor agonist, is considered to have the potential to reduce PONV [20]. In a study on abdominal surgery, the incidence of PONV with oliceridine was significantly lower than that with sufentanil [21]. Therefore, we aim to investigate the efficacy of oliceridine in reducing PONV in thyroid surgery.

In this study, the drug administration regimen was set as follows: sufentanil 0.3 μg/kg or oliceridine 0.06 mg/kg was administered during anaesthesia induction, and sufentanil 0.15 μg/kg or oliceridine 0.03 mg/kg was supplemented every 15 min during the surgery. The determination of this regimen was based on the following considerations: first, according to FDA official package insert, the efficacy of 1 mg of oliceridine is approximately equivalent to 5 mg of morphine [15]. Based on this conversion, the equivalent ratio of sufentanil to oliceridine is 1:200 approximately [11–15]. Based on this ratio, we set the dose ratio of sufentanil to oliceridine as 1:200, so that the morphine equivalents in the two groups were similar. Second, compared to visceral surgeries, the pain stimulation during thyroid surgery is mild relatively [22–25]. So, based on previous studies, we employed intermittent additional doses rather than continuous infusion for intraoperative analgesia. Third, no other opioids were used during the perioperative period to avoid influencing the outcome indicators. Fourth, through our own practice, this regimen has been confirmed to meet the analgesic requirements of thyroid surgery. As a result, the intraoperative morphine equivalents consumed in both groups were approximately 60 mg, which is higher than the consumption reported previously [1]. Although this amount is sufficient to meet the analgesic requirements for thyroid surgery, we acknowledge that it may exaggerate the between-group differences and limit the external validity of our findings, given the current preference for opioid-sparing regimens.

The most critical finding of this study is that oliceridine reduces the incidence of PONV while providing analgesic effects comparable to sufentanil. All patients received prophylactic dexamethasone 5 mg plus ondansetron 4 mg perioperatively, yet a significant between-group difference in PONV was still observed, further highlighting the significant effect of oliceridine in reducing PONV. To date, the mechanism by which oliceridine alleviates PONV remains unclear. Current research suggests that, unlike traditional opioids, oliceridine exhibits high selectivity for G-protein-coupled receptor-mediated signalling pathways and reduces the recruitment of β-arrestin to opioid receptors phosphorylated by G-protein-coupled receptor kinases [26]. β-arrestin can form complexes with phosphorylated opioid receptors, blocking G-protein coupling and reducing the analgesic efficacy of the drug; β-arrestin also mediates opioid-related adverse effects such as PONV through pathways including the activation of mitogen-activated protein kinases [27]. Therefore, this bias towards G-protein-coupled receptor signalling, coupled with reduced β-arrestin recruitment, likely represents the primary mechanism underlying oliceridine’s beneficial effect on PONV. Further studies with relevant biomarker detection are warranted to validate these hypotheses. Besides, given that the conversion ratio between sufentanil and oliceridine (1:200) used in this study has not been widely validated, caution is still needed when comparing the difference in PONV incidence, because if oliceridine was underdosed relative to sufentanil, the lower PONV could simply reflect lower opioid exposure rather than receptor bias.

Surprisingly, there was no significant difference in postoperative pain scores between the two groups. Nevertheless, a prudent comparison of the analgesic efficacy of oliceridine and sufentanil is still necessary, considering that sufentanil is a well-established potent analgesic. No difference in analgesic efficacy was observed, possibly due to a floor effect of the NRS score. Specifically, the mean postoperative NRS scores in both groups ranged only from 1.0 to 1.6, indicating mild pain after thyroid surgery. This low baseline pain limited the statistical power to detect subtle differences in analgesic potency between the two opioid drugs. Currently, few studies have compared the two for intraoperative analgesia, with most focusing on their effects in postoperative analgesia [28–30]. In surgical procedures involving more severe pain, such as gastrointestinal surgery, thoracic surgery, and cardiac surgery, no studies have yet confirmed that oliceridine can achieve analgesic effects comparable to those of sufentanil. Furthermore, from a pharmacological mechanism perspective, oliceridine lacks agonistic effects on the κ receptor, so its sole use may not meet analgesic requirements in certain surgeries [31,32].

In terms of other adverse reactions, and consistent with the reduction in PONV, exploratory analysis showed that patients treated with oliceridine had a lower incidence of abdominal distension, potentially suggesting a milder gastrointestinal impact compared with sufentanil. The incidence of respiratory depression was low in both groups, possibly due to the relatively young age of the enrolled population, as the incidence of respiratory depression is lower in younger individuals than in the elderly; therefore, no difference between the two groups was observed, given the limited sample size.

This study has the following limitations: First, it was a single-center study with a small sample size; future multicentre, large-sample clinical studies are needed to further validate the analgesic efficacy and adverse effects of oliceridine. Second, multiple secondary outcomes were tested without adjustment for multiple comparisons, which may increase the risk of type I error. Third, perioperative blood or urine samples were not collected, preventing investigation of the molecular mechanisms underlying the reduction in PONV with oliceridine. Fourth, the level of morphine equivalent consumption in this study was elevated, which could overstate the differences between groups and constrain the generalisability of our results.

## 5. Conclusion

For young ASA I–II patients undergoing thyroid surgery, oliceridine yields adequate postoperative analgesia and lower PONV rates versus sufentanil. Given the mild postoperative pain observed in this low-pain thyroid cohort, additional trials involving high-intensity surgical procedures are needed to confirm consistent equivalence.

## Supplementary Material

CONSORT Checklist.doc

## Data Availability

The datasets used and/or analysed during the current study are available from the corresponding author on reasonable request.

## Open scholarship

This article has earned the Center for Open Science badges for Preregistered. The meterials are openly accessible at https://www.chictr.org.cn/showprojEN.html?proj=268261.
